# Evaluation of the effect of osseodensification on the peri-implant condition: A clinical, biochemical, and radiographic trial

**DOI:** 10.34172/joddd.025.42046

**Published:** 2025-09-30

**Authors:** Ahmed Ali Elgaddah, Ibrahim Hammad Ibrahim, Asem Mohamed Kamel, Khalid Seddik Hassan

**Affiliations:** Department of Oral Medicine, Periodontology, Oral Diagnosis, and Dental Radiology, Faculty of Dental Medicine, Al-Azhar University, Assiut Branch, Asyut Governorate, Egypt

**Keywords:** Bone density, IL-6, Implant, Osseodensification, VEGF

## Abstract

**Background.:**

This trial aimed to compare osseodensification with traditional implant site preparation in terms of clinical outcomes, radiographic findings, interleukin-6 (IL-6), and vascular endothelial growth factor (VEGF) levels in the peri-implant sulcus.

**Methods.:**

Sixteen patients were randomly assigned to two groups. In group 1, eleven sites received a small-diameter implant according to the conventional method; in group 2, eleven sites received an implant after osseodensification. The modified plaque index, modified bleeding (sulcus) index, and peri-implant probing depth were recorded for all patients on the day of implant placement (baseline) as well as at 3 and 6 months. The preoperative and postoperative alveolar ridge widths were measured, and the marginal bone loss (MBL) around the implant was assessed. Gingival crevicular samples were assayed using ELISA.

**Results.:**

For the MPI, mSBI, and PPD, no statistically significant differences were reported across the groups at baseline and 3 and 6 months. Group 2 showed a lower marginal bone level and higher bone density, lower VEGF, and lower IL-6 levels than group 1.

**Conclusion.:**

Osseodensification was shown to preserve bone and augment the ridge width, unlike conventional osteotomy with a small-diameter implant. The association of VEGF and IL-6 may be used as a marker for bone resorption and revascularization around dental implants.

## Introduction

 Tooth decay is a debilitating and irreversible condition, described as “the ultimate sign of oral disease risk.” Despite a drop in the past ten years in the rate of complete tooth missing, edentulism is still a serious condition throughout the globe, especially among older adults. Tooth loss can lead to immediate disability, functional morbidity, and physical, psychological, and gregarious impairments.^1^ Consequently, the primary goals of prosthetic or implant-supported therapy are to maintain the patient’s beauty and well-being while also restoring function, including speech and mastication.^2^ A new technique for biomechanical bone preparation for dental implants is called osseodensification, which has been developed over the past decade.^3^ It is a biomechanical bone preparation maneuver used to place a dental implant using specially designed condensing instruments called Densah® burs (Versah® LLC, MI, USA).^4^ Osseodensification aids in ridge expansion while maintaining the integrity of the alveolar bone, enabling accurate implant placement in the autogenous alveolar site and achieving sufficient primary stability. Osseodensification facilitates the preservation of bone mass and speeds up the transition to the restorative phase.^5^ Interleukin-6 (IL-6) is a pro-inflammatory mediator that coordinates immunological reactivity and hemopoietin. It is secreted by immune cells, adipose tissue, and muscles with a significant influence on the inflammatory process.^6^ Vascular endothelial growth factor (VEGF), among other factors, plays a role in the angiogenesis and osteogenesis of bone healing following implantation. One of these factors, VEGF, is a significant stimulator and is essential for bone healing, although its effect on dental implants remains unclear.^7^ The null hypothesis was that there are no additional benefits to using the osseodensification technique. Hence, the current study compared osseodensification with traditional implant site preparation in terms of the clinical outcomes, radiographic findings, and IL-6 and VEGF levels in the peri-implant sulcus.

## Methods

###  Study setting and population

 This randomized clinical trial was conducted on 16 patients of both sexes, aged 39‒59, who had missing maxillary teeth and were interested in having dental implants. The Helsinki Declaration ethical guidelines were followed. Patients were chosen from the Department of Oral Diagnosis and Dental Radiology, Outpatient Clinic of Oral Medicine, Periodontology, and Al-Azhar University’s College of Dentistry (Assiut branch). The Ethics Committee of the Faculty of Dentistry, Al-Azhar University, approved the study under the code AUAREC20230001-1. This study has been archived in the ClinicalTrials.gov Protocol Registration and Results System with the ID NCT06689969. Before beginning the work, verbal and written consent were obtained from the participants.

###  Sample size calculation and power analysis 

 The G*Power system software program (G*Power, Ver. 3.1.9.6, copyright 1992–2020, Franz Faul, University of Kiel, Kiel, Germany) was used to conduct the power analysis for sample size calculation. α = 0.05 (type I error) and β = 0.20 (type II error) were defined as the significance thresholds to detect a significant difference (ƍ) in the implant stability quotient among studied groups when primary implant stability was used as the principal aggregate variable, with a 90% confidence interval. Consequently, the compulsory sample scale for this inquisition was set to 16 patients, with 90.38% actual power.

###  Random allocation and blindness

 The participants were randomly classified into equal groups using the coin-flipping technique. Single oblivion was designed for outcome assessments.

###  Grouping and selection benchmarks

 The inclusion criteria were as follows: individuals aged > 18 who had thin ridges and missing bilateral maxillary posterior teeth,^8^ with no systemic diseases. The exclusion criteria included cases with severe skeletal discrepancy, patients with parafunctional habits, those who had a history of lost implants in the prospective implant surgery area, smokers, those who had undergone radiation for head and neck cancers, those receiving chemotherapy, those with systemic conditions such as hypertension, diabetes, blood disorders, metabolic bone disorders, liver disease, and renal diseases, those who were immunocompromised, and those with a lack of compliance to oral hygienic homecare.^9^ Forty-eight implants were placed in the selected sites. In group 1, eight patients with missing teeth and a narrow ridge received 24 small-diameter implants using the conventional method. In group 2, eight patients with missing teeth and a narrow ridge received 24 implants using the osseodensification technique with a Densah bur.

###  Preoperative preparation

 Each case was examined using Cone beam computed tomography (CBCT) to determine the supporting bone characteristics, measure the height and breadth of the ridge, and identify the main anatomic characteristics. To create an oral environment more conducive to wound healing, all patients underwent full-mouth phase I periodontal therapy and received training on basic oral hygiene. Amoxicillin trihydrate, 1 g in a single dosage, was prescribed as preventive antibiotic treatment the day before surgery. In addition, before surgery, 0.02% chlorohexidine HCl mouthwash was administered.^10^

###  Conventional surgical technique

**Figure 1:**
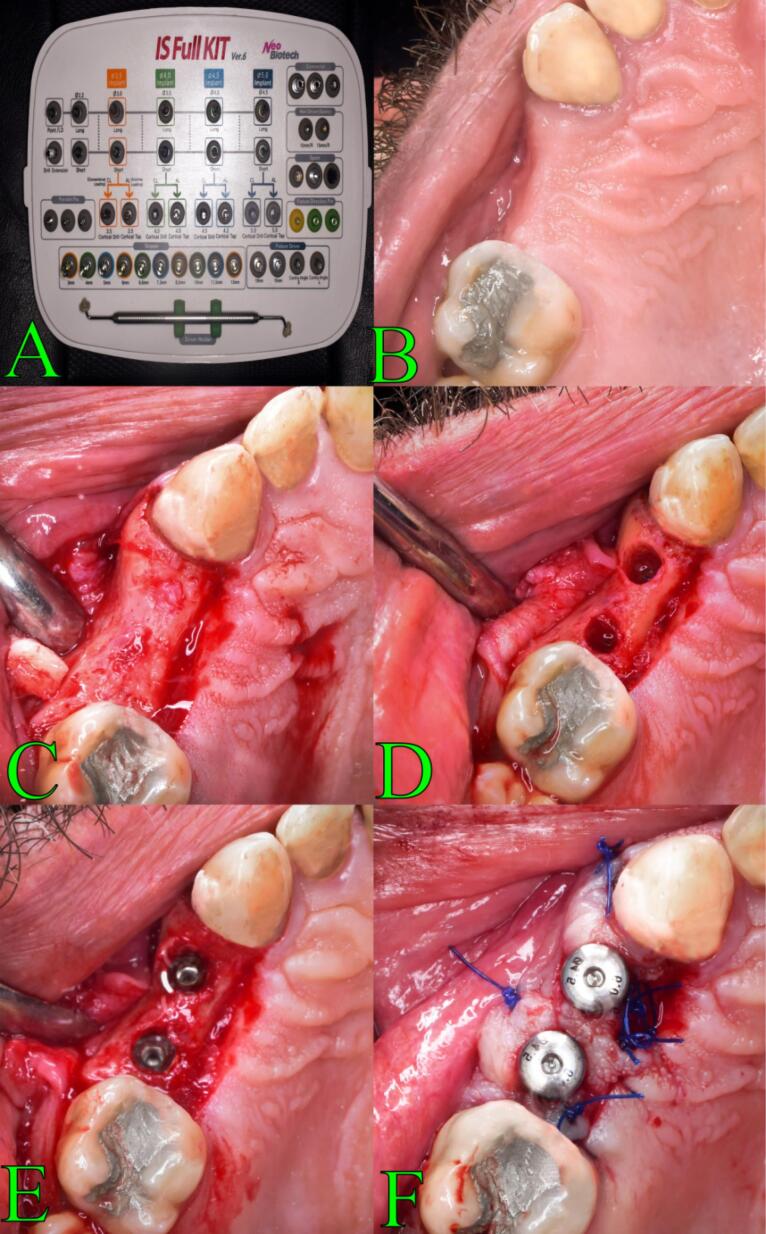
Conventional surgical technique

 The surgical site was anesthetized, and a full-thickness flap was reflected after a crestal incision had been performed. Site marking was the first step in preparing the place for implantation. Subsequently, a pilot drill was revolved at 1200 rpm in a clockwise rotation to the desired depth, creating a 1.5-mm first pilot osteotomy. An x-ray was obtained using paralleling pins to validate the angle between the surrounding teeth and the implants. Eventually, the implant’s precise placement was established. To prepare the osteotomy site to the desired diameter, drills were used sequentially at 1200 rpm in a clockwise motion. Gradually, larger drill diameters were used for incremental drilling. Drill sizes were used in ascending order with justification for the required implant diameter (Figure 1).

###  Osseodensification surgical technique

**Figure 2:**
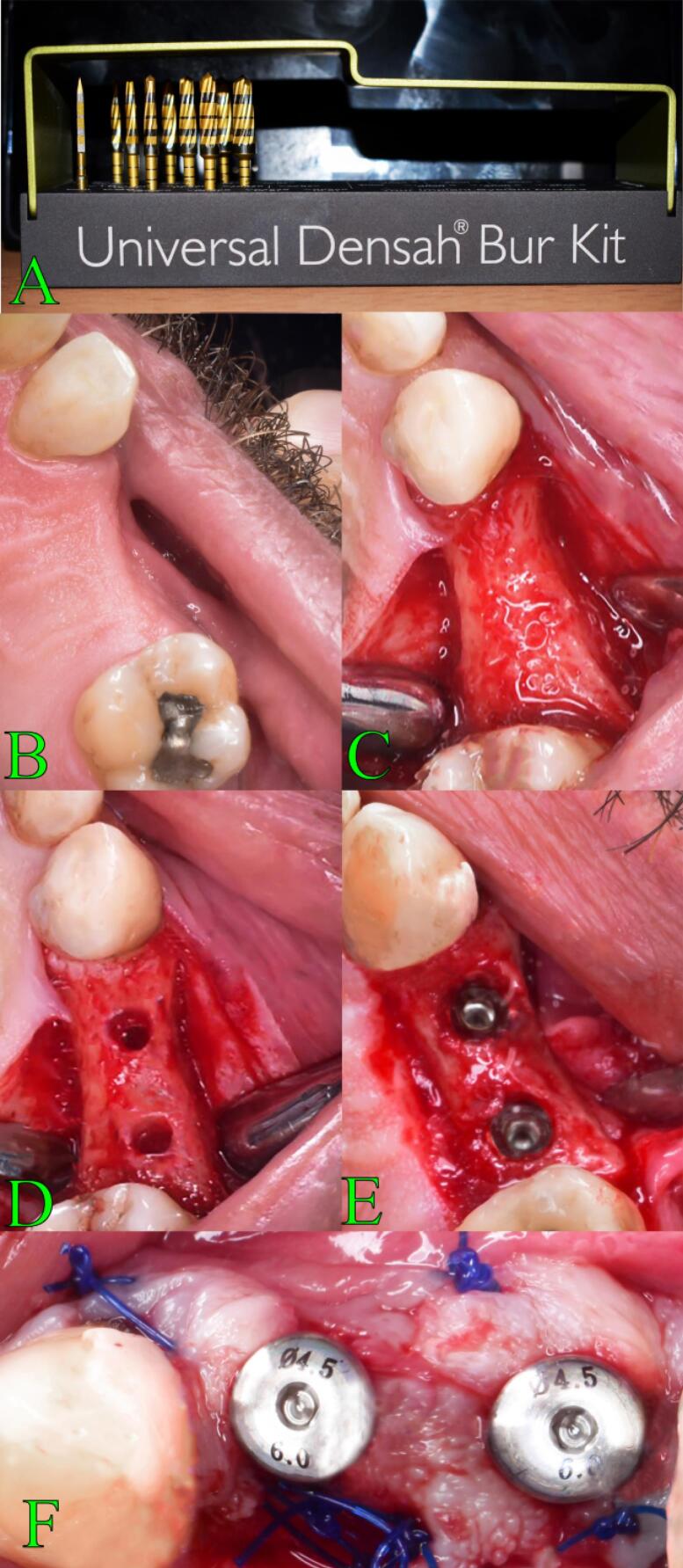
Osseodensification surgical technique

 Beginning with site marking, the area was prepared for implantation. A high-speed 1/20 surgical handpiece and implant motor (Surgic Pro®, NSK, Japan) were then used to construct the initial pilot osteotomy using a pilot drill spun at an adjusted rpm in a clockwise spinning mode to the desired profundity. To verify the angle between the surrounding teeth and the implants, paralleling pins were used to capture an x-ray. After it was determined that the implant was in the proper location, osseodensification was used to extend the osteotomy using a Densah® Bur VT1525 2.0 mm (Versah^TM^, LLC, USA) in a lack-cutting anticlockwise spinning mode at an adjusted rpm (Densifying Mode). The osteotomy was expanded to the desired diameter by repeatedly using a Densah^TM^ bur operating in an anticlockwise (CCW) direction at an adjusted rpm (Densifying Mode). Gradually, increasing drill diameters were used for incremental drilling. Drill sizes were used in ascending order with justification for the required implant diameter (Figure 2).

###  Implant placement 

 Tapered implants (Neobiotech^®^, Neobiotech Co., Ltd., Korea) were carefully screwed and seated into the prepared site, with all threads buried, according to the manufacturer’s instruction. The platform was flushed at the crestal bone to achieve initial stability for the implants. The primary stability of the implants was then assessed before they were fastened to healed abutments.

###  Postoperative instructions and medications 

 Augmentin 1-g tablets were prescribed for each patient twice daily for 5 days. Analgesics and anti-inflammatory agents were prescribed as follows: Brufen® 400 mg TDS for 5 days was prescribed. For oral hygiene, a soft toothbrush was suggested. Additionally, the participants were asked to follow a soft diet to avoid any damage to the gingival tissue surrounding the implant sites during the first few weeks. Patients underwent weekly checkups for the first three weeks following surgery, followed by visits at 1-, 3-, and 6-month intervals. Sutures were removed after 7 to 10 days.

###  Periodontal evaluation 

 The modified plaque index, modified bleeding index, and peri-implant probing depth were documented for all patients on the day of the implant placement and at 3 and 6 months using a UNC-15 periodontal probe, graded in mms.^11^

###  Implant primary stability 

 An Osstell® tip of a Mentor magnetic resonance device (Osstell; Integrated Diagnostics Ltd., Göteborg, Sweden), which employs resonance frequency analysis to determine fixture stability, was handled to check the initial stability of each implant.

###  Radiographic evaluation 

**Figure 3:**
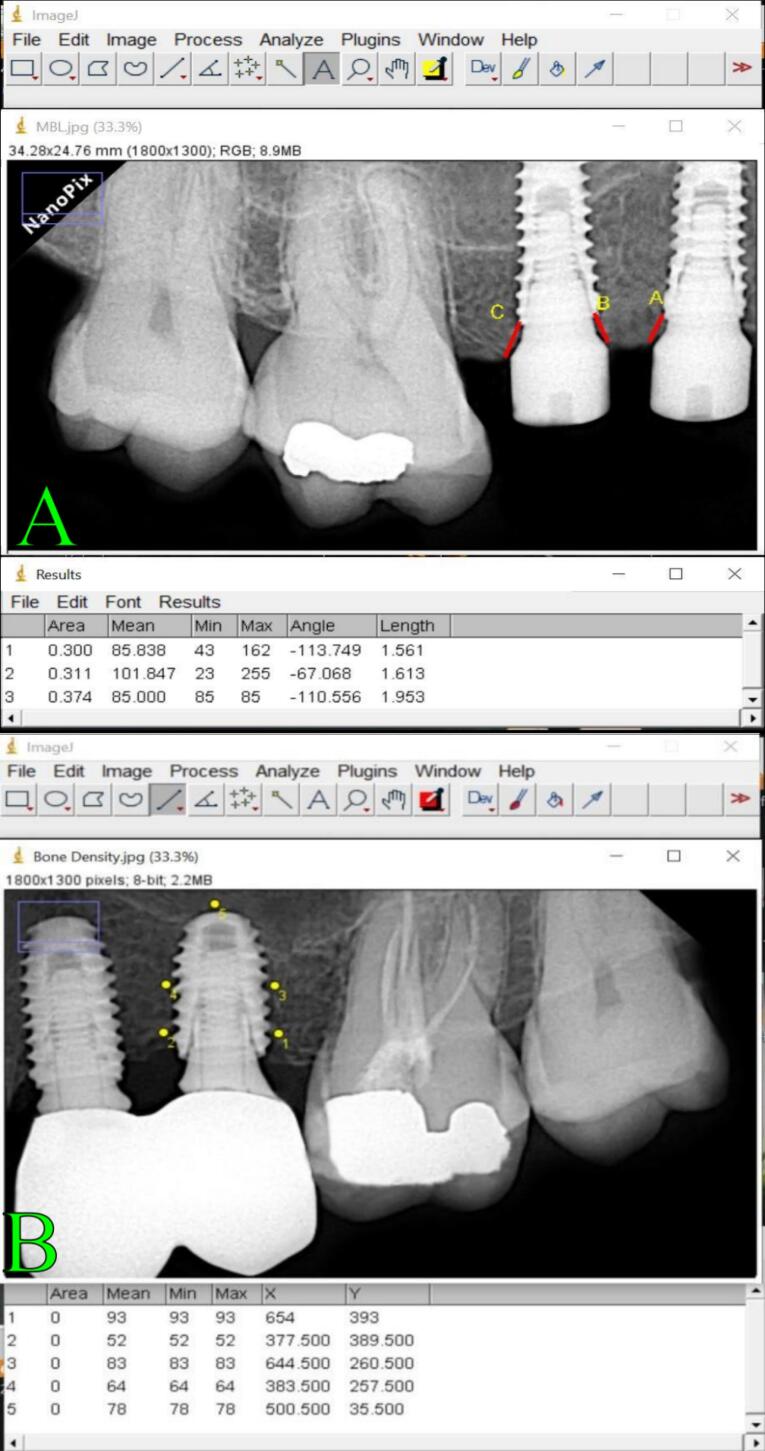
Radiograph evaluation of bone density and marginal bone loss

 The bone density (BD) was measured using the “ImageJ” computerized application (1.51n; Wayne Rasband, National Institute of Health, Bethesda, Maryland); the mean gray values (average intensity is ascertained on a scale of 0 to 256, where 256 (8 bits) reflects the nature of whitest pixels on the object, and 0 value represents the darkest pixel areas on the object) of specific areas on various digital radiographic images taken during the postoperative course were measured to evaluate the bone density in the bone around the implants^12^ (Figure 3A).

 The marginal bone loss (MBL) was measured using periapical radiographs, which were taken at the beginning and 1, 3, and 6 months following implant insertion. Radiographs were obtained using a digital sensor and the PRO 70X Intra radiography device, which operates at 70 kVp and 8 mA (Figure 3B).

###  Biochemical evaluation 

 The IL-6 and VEGF in peri-implant sulcular fluid samples were assessed. A highly sensitive ELISA gadget (Quantikine HS ELISA Human IL-6 (HS600B, R&D Systems, Minneapolis, MN) and VEGF ELISA kit (ab100662; Abcam, Cambridge, UK) were used to detect the IL-6 and VEGF levels in pg/mL in gingival crevicular exudate according to the manufacturer’s instructions.

###  Prosthetic procedure 

 Three months later, an optical impression was taken using a scan body and scanner to fabricate the final zirconia restoration, which was then cemented onto the abutment.

###  Statistical analysis

 The data were collected and organized, and statistical analysis was performed using the latest version of the International Business Machines (IBM)® Statistical Package for SPSS. The significance level was set at *P* ≤ 0.05. The data were reported as mean and standard deviation values and regularity was assessed using the Kolmogorov–Smirnov and Shapiro–Wilk tests. MPI and MGI values appeared in non-parametric (not-normal) dispersal (scores), while the remaining values of other indicators appeared in parametric (normal) distribution. For the non-parametric values, the Mann–Whitney test was used to compare two entities with unrelated values, and the Friedman test was used to compare more than two groups. The Wilcoxon test was used to differentiate between the two entities in related values. For the parametric data, the independent sample t-test was used to compare two entities in non-related values. Repeated-measures ANOVA was used to compare more than two time intervals. A dependent sample t-test was used to compare two entities. Spearman’s correlation was used to investigate the correlation between distinct variables.

## Results

 These investigations were conducted on 16 individuals (nine females and seven males) who suffered from missing posterior teeth with a narrow ridge, seeking implant placement, aged 39‒59, with a mean age of 49.3 ± 5.6 years. Forty-eight implants were placed, with diameters ranging from 3.1 to 4.6 mm. The linear measure of implant length varied from 9 to 11 mm. The implant width was determined according to the amount of achievable buccal/lingual augmentation after the disparate preparation procedure, conserving 1 mm of buccal/lingual cortical plate around the fixtures. The length was based on the pre-surgical assessments of the vertical height of the alveolar ridge, indorsing at least 1 mm from anatomical structures as a refuge distance.

 Modified plaque index (MPI): There was no statistically significant difference between groups 1 and 2 at baseline and after 3 and 6 months (*P* = 1, *P* = 0.427, and *P* = 0.345, respectively).

 Modified sulcus bleeding index (MSBI): There was no statistically significant difference between groups 1 and 2 at baseline and after 3 and 6 months (*P* = 1, *P* = 0.289, and *P* = 0.544, respectively).

 Peri-implant probing depth: There were statistically significant differences between groups 1 and 2 at baseline and after 6 months (*P* < 0.001 and *P* < 0.001, respectively). There was no statistically significant difference between groups 1 and 2 after 3 months (*P* = 0.100) (Table 1).

**Table 1:** The mean and standard deviation (SD) values of the modified plaque index, modified bleeding index, and the peri-implant probing depth of both groups

	**Group 1**		**Group 2**		***P*** **-value**
	**Mean**	**SD**	**Mean**	**SD**	
MPI					
Baseline	0.00	0.00	0.00	0.00	1^ns^
After 3 months	0.13	0.09	0.17	0.09	0.427 ^ns^
After 6 months	0.24	0.16	0.182	0.07	0.345 ^ns^
P-value	< 0.001*		0.001*		
mSBI					
Baseline	0.00	0.000	0.00	0.00	1 ^ns^
After 3 months	0.19	0.10	0.14	0.10	0.28 ^ns^
After 3 months	0.20	0.07	0.16	0.10	0.54 ^ns^
*P* value	0.00 *		< 0.00*		
Peri-implant probing depth					
After 3 months	2.44	0.28	2.39	0.3	0.1^ns^
After 3 months	2.82	0.21	2.09	0.28	< 0.00*
*P* value	0.00*		< 0.00*		

MPI: modified plaque index, MSBI: modified sulcus bleeding index, ns: Non-significant (*P* > 0.05). * Significant (*P* < 0.05).

 Implant stability quotient (ISQ): Differences between the groups were significant as osseodensification resulted in higher implant stability quotient values than implants placed using traditional techniques (Table 2).

**Table 2:** The range, minimum, maximum, mean ± SD, and unpaired t-test used to compare the ISQ between groups with a statistically significant difference*

	**Range**	**Minimum**	**Maximum**	**Mean**	**SD**
Group 1	20	55	66	61.45	5.22
Group 2	9	66	77	69.79	2.84
**Unpaired t-test**					
		**t**		***P*** ** value**	
G 2 vs. G 1		2.92		0.015*	

 Marginal bone level (MBL): At baseline and 6 months, group 2 showed a lower marginal bone level than group 1.

 Bone density (BD): At baseline, group 2 exhibited a higher bone density than group 1. There was no statistically significant difference between groups 1 and 2 at 3 and 6 months (*P* = 0.050 and *P* = 0.332, respectively) (Table 3).

**Table 3:** The mean and standard deviation (SD) values of the MBL and bone density of diverse groups

	**Group 1**		**Group 2**		***P*** ** value**
	**Mean**	**SD**	**Mean**	**SD**	
MBL					
Baseline	0.36	0.16	0.29	0.15	1^ns^
After 3 months	0.49	0.15	0.17	0.25	< 0.00*
After 3 months	0.63	0.24	0.77	0.13	0.12^ns^
*P* value	< 0.001*		< 0.001*		
Bone density					
Baseline	101.62	7.555	122.35	7.71	< 0.00*
After 3 months	107.37	7.588	100.66	6.48	0.05^ns^
After 3 months	121.5	6.623	118.74	5.71	0.33^ns^
*P* value	< 0.00*		< 0.00*		

*Note*: ANOVA was used to compare different intervals within groups, and the unpaired t-test was used to compare groups. * Significant (*P* < 0.05), ns: non-significant (*P* > 0.05).

 The VEGF showed the following results at baseline and after 1, 3, and 6 months: *P <*0.001, *P <*0.001, *P <*0.001, and P = 0.004, respectively. Group 2 exhibited a lower VEGF than group 1 (Table 4). The IL-6 levels at baseline and after 1, 3, and 6 months were significantly different (*P <*0.001).

**Table 4:** The mean and standard deviation (SD) values of the VEGF and IL-6 of both groups in the pictogram

	**Group 1**		**Group 2**		***P*** ** value**
	**Mean**	**SD**	**Mean**	**SD**	
VEGF					
Baseline	815.46	58.072	664.49	51.01	< 0.00*
After 1 month	420.44	25.46	795.77	47.27	< 0.00*
After 3 months	781.05	60.24	628.61	21.64	< 0.00*
After 6 months	595.96	39.36	642.53	21.01	0.00*
*P* value	< 0.00*		< 0.00*		
IL-6					
Baseline	141.14	3.99	57.78	4.98	< 0.00*
After 1 month	70.53	4.1	151.35	7.9	< 0.00*
After 3 months	125.02	7.41	69.95	5.28	< 0.00*
After 6 months	85.31	7.7	87.53	7.6	0.52ns
*P* value	< 0.00*		< 0.00*		

Note: ANOVA was used to compare different intervals within groups, and the unpaired t-test was used to compare the groups. * Significant (*P* < 0.05), ns: non-significant (*P* > 0.05).

###  Correlation between the different parameters

 Concerning the MPI correlation results, the MPI parameter exhibited a positive correlation with all parameters except the VEGF, which showed a negative correlation with MPI. The strongest correlation was found with the MBI, while no correlation was observed with IL-6. The MBI correlation results revealed a positive correlation with all the parameters, except with the VEGF, which showed a negative correlation. The strongest correlation was found with the MPI, while the weakest correlation was observed with IL-6. The PPD correlation results showed a positive correlation with all the parameters; the strongest correlation was observed with IL-6, while the weakest correlation was found with MBL and bone density. The MBL correlation results showed a positive correlation with all the parameters, except with VEGF and IL-6, which exhibited a negative correlation. The strongest correlation was observed with the VEGF, while the weakest correlation was found with the PPD. The bone density correlation results showed a positive correlation with all the parameters except VEGF and IL-6, which exhibited a negative correlation. The strongest correlation was found with the MBL, while the weakest correlation was observed with the PPD. The VEGF correlation results revealed a negative association with MPI, MBI, MBL, and bone density, with a positive correlation with PPD and IL-6. The strongest correlation was observed with IL-6, while the weakest correlation was found with bone density. The IL-6 correlation results revealed a negative association with MBL and bone density, with a positive correlation with MPI, MBI, PPD, and VEGF. The strongest correlation was observed with the VEGF, while the weakest correlation was found with the MPI (Table 5).

**Table 5:** The correlation between the studied groups regarding the different parameters

		**MPI**	**MSBI**	**PPD**	**MBL**	**Bone density**	**VEGF**	**IL-6**
MPI	*r*		0.67	0.29	0.53	0.17	-0.35	0.005
	*P*		0.00^*^	0.02^*^	0.00^*^	0.18	0.00^*^	0.97
MGI	*r*	0.675		0.51	0.47	0.21	-0.15	0.11
	*P*	0.00^*^		0.00^*^	0.00^*^	0.09	0.23	0.38
PPD	*r*	0.29^*^	0.51		0.06	0.06	0.32	0.52
	*P*	0.02^*^	0.00^*^		0.6	0.6	0.01^*^	0.00^*^
MBL	*r*	0.053	0.476	0.06		0.35	-0.59	-0.18
	*P*	0.00^*^	0.00^*^	0.6		0.00^*^	0.00^*^	0.15
Bone density	*r*	0.17	0.21	0.06	0.35		-0.14	-0.26
	*P*	0.18	0.09	0.6	0.00^*^		0.27	0.03^*^
VEGF	*r*	-0.35	-0.15	0.32^*^	-0.59^*^	-0.14		0.67
	*P*	0.00^*^	0.23	0.02	0.00	0.27		0.00^*^
IL-6	*r*	0.00	0.11	0.52^*^	-0.18	-0.26	0.67	
	*P*	0.97	0.38	0.00	0.15	0.03^*^	0.00^*^	

r: degree of correlation, P: degree of significance, -: negative correlation, MPI: modified plaque index, MSBI: modified sulcus bleeding index, PPD: peri-implant probing depth.

## Discussion

 Bone deformity and atrophy, especially on the buccal aspect of the jaw, often occur concomitant with tooth extraction, leading to a narrow ridge. Some guidelines suggest that a 1.5‒2-mm bony zone around the implant should be kept to prevent postoperative bone resorption.^13^ To resolve the narrow ridge situation, alveolar ridge augmentation has been performed using many methods. One of these methods is osseodensification. The superiority of this maneuver lies in its ability to simultaneously expand the ridge and place implants in a series of narrow ridges.^14^ This surgical study aimed to evaluate the effectiveness of osseodensification versus traditional implant site preparation in terms of clinical and radiographic findings, as well as IL-6 and VEGF levels in the peri-implant sulcus. The modified sulcus bleeding index (mSBI) was used in the current investigation as a clinical indicator of the presence or absence of inflammation. Bleeding on probing (BOP) was a predictor of stable peri-implant conditions since it had a strong negative predictive value.^15^ This finding was consistent with research indicating that healthy sites exhibited no bleeding (0%), whereas both peri-implant mucositis and peri-implantitis sites had remarkably elevated BOP (67% and 91%, respectively).^16^ The peri-implant pocket depth (PPD) examination results indicated positive results throughout the evaluation process. This study revealed a significant difference in PPD between the two groups, with the mean (PPD) in group 1 being 2.44 ± 0.28 at 3 months, which changed to 2.82 ± 0.21 at 6 months, while in group 2, it was 2.82 ± 0.21 at 3 months, which then decreased to 2.09 ± 0.28 at 6 months. The current findings indicated no significant differences between the two groups with regard to limited plaque buildup around the implant margins and good oral hygiene habits among patients in all groups during the observation period. This finding is consistent with other studies that indicate patient oral hygiene and the management of plaque buildup play a major role in determining implant success or failure.^17^ MBL surrounding dental implants is viewed as a severe issue, and substantial bone loss has long been considered one of the major causes of implant failure.^18^ Levels of MBL in this current trial, in accordance with a widely acknowledged standard, were examined; a success criterion of implant surgical procedures should show minimal bone loss during the first year (1.5 mm); then 0.2 mm yearly can be tolerated.^19^ The study’s findings, which indicated that this type of preparation appeared to exert strain on the crestal cortex bone and cause a large MBL, can be used to explain why group 2 showed more MBL than group 1 at three months.^20^ The results of the present scrutiny regarding implant stability were similar to those of Stavropoulos and colleagues’study,^21^ which found that implants inserted using the bone condensation approach had high primary stability; however, the alveolar ridge had small fissures coronally around the collar of the implant. In addition, Huwais and Meyer^22^ stated that osseodensification resulted in a compression of bone along the osteotomy depth and enhanced the mineral bony density along the osteotomy’s outsider periphery. Moreover, a reverse compression of the bone tissue against the implant body was formed due to the spring-back action caused by the elastic strain recovery of the compressed bone, strengthening the implant’s main stability. In this appraisal of bone density, the current values were consistent with those reported by Trisi et al,^23^ who observed a 30% increase in bone volume percentage, ridge width, and density in the osseodensification group. The coronal implant site, where the bone trabeculae were enlarged due to the inclusion of autogenous bone fragments during healing, demonstrated the highest increase in bone density in the osseodensification area. Higher concentrations of VEGF and IL-6 were found in the GCF of patients with conventionally placed implants, especially at baseline, compared to those placed with osseodensification. The explanation for these results, as mentioned by Lahens et al,^24^ may be due to the osteotomy site being prepared using conventional drills that excavate bone and establish a good blood supply, allowing access to anti-inflammatory agents and growth factors. In the study by Dipalma et al^25^ and the review by Insua et al,^26^ it was reported that the osteotomy site was prepared using Densah drills that densified the bone and thus reduced the blood supply. At 1 month, higher concentrations of VEGF and IL-6 were found in the GCF of patients with osseodensification compared to the conventionally prepared sites. Osseodensification can lead to a decreased blood supply due to the elevated density at the osteotomy site, as addressed by Gandhi et al.^27^ In their histomorphometric review of microvessel density, Bian et al^28^ noted a strong relationship between VEGF expression and revascularization. The present feedback is in accordance with the Lauritano et al^29^ trial that determined a strong positive correlation between the basal production of IL-6, IL-1, and VEGF in human pituitary tumors. One limitation of the current trial was the lack of histological analysis of peri-implant tissue. Therefore, in vitro studies are recommended. Studies with large sample sizes involving other biochemical mediators of bone metabolism should also include the long-term consequences of implant follow-up.

## Conclusion

 The implant survival rate suggested that the conventional surgical technique with a narrow-diameter implant and the osseodensification surgical technique can be considered treatments with promising survival rates in a narrow alveolar ridge. Osseodensification has been shown to preserve bone and augment the ridge width, unlike conventional osteotomy. The association of VEGF and IL-6 may be used as a marker for bone resorption and revascularization around dental implants, respectively.

## Competing Interests

 The authors declare no conflicts of interest.

## Ethical Approval

 The study was approved by the Ethics Committee, Faculty of Dental Medicine, Al-Azhar University with the number AUAREC20230001-01.

## Informed Consent

 The participants provided informed consent.
